# Motion Event Similarity Judgments in One or Two Languages: An Exploration of Monolingual Speakers of English and Chinese vs. L2 Learners of English

**DOI:** 10.3389/fpsyg.2017.00909

**Published:** 2017-06-07

**Authors:** Yinglin Ji

**Affiliations:** Research Centre for Language and Cognition, Shenzhen UniversityShenzhen, China

**Keywords:** linguistic relativity, spatial cognition, similarity judgment, reaction time

## Abstract

Languages differ systematically in how to encode a motion event. English characteristically expresses manner in verb root and path in verb particle; in Chinese, varied aspects of motion, such as manner, path and cause, can be simultaneously encoded in a verb compound. This study investigates whether typological differences, as such, influence how first and second language learners conceptualize motion events, as suggested by behavioral evidences. Specifically, the performance of Chinese learners of English, at three proficiencies, was compared to that of two groups of monolingual speakers in a triads matching task. The first set of analyses regarding categorisation preferences indicates that participants across groups preferred the path-matched (rather than manner-matched) screens. However, the second set of analyses regarding reaction time suggests, firstly, that English monolingual speakers reacted significantly more quickly in selecting the manner-matched scenes compared with monolingual speakers of Chinese, who tended to use an approximately equal amount of time in making manner- and path-matched decisions, a finding that can arguably be mapped onto the typological difference between the two languages. Secondly, the pattern of response latency in low-level L2 learners looked more like that of monolingual speakers of Chinese. Only at intermediate and advanced levels of acquisition did the behavioral pattern of L2 learners become target-like, thus suggesting language-specific constraints from the L1 at an early stage of acquisition. Overall, our results suggest that motion event cognition may be linked to, among other things, the linguistic structure of motion description in particular languages.

## Introduction

The main arguments of the linguistic relativity hypothesis, also known as the ‘Sapir-Whorf hypothesis’ (Whorf, 1956), focus on how properties of a given language influence the structure and content of thought, thus affecting the way that humans view reality. As later illustrated by Slobin (1996), one’s native language is not a neutral coding system of an objective reality; Instead, it trains its speakers, from childhood, to pay habitual attention to specific dimensions of experience that are already enshrined in grammatical categories. Seen in this way, acquiring a native language entails learning a particular way of thinking (‘Thinking for speaking’ hypothesis in Slobin, 1996, p. 76–89). Such an effect of linguistic relativity has been reported in some investigations conducted across different groups of language speakers, and in varied domains, such as color perception, object categorisation, temporal representation, space and motion events (see, for instance, Boroditsky, 2001; Levinson, 2003; Hohenstein, 2005; Regier and Kay, 2009; Lupyan, 2012; Zlatev and Blomberg, 2015). Given that a particular language instantiates a special way of thinking, and that differences in linguistic structure foster variations in cognitive pattern, one naturally wonders what would happen to people who have two or more languages at their disposal? Do they look at the world differently from monolingual speakers? The linguistic relativity hypothesis can thus have far-reaching consequences for a number of important issues in second language (L2) acquisition, which include the following questions: to what extent does an L2 learner recalibrate his cognitive dispositions as a result of additional language learning? What is the nature of the dynamic relationship between progress in L2 acquisition and the shifting cognitive state of an L2 speaker? What are the (extra)linguistic factors that determine the language-specific cognitive behavior of an L2 speaker in non-verbal tasks (as discussed in Bylund and Athanasopoulos, 2014, p. 953–954).

In this context, the present study investigates how Chinese adult L2 learners of English at different proficiencies conceptualize complex motion events (i.e., caused motion involving path, causality and varied types of manner information) in a triads matching task. Their performance is then compared to that of Chinese and English monolingual speakers with the aim of shedding fresh light on the question of linguistic relativity recast in an L2 context: the extent to which non-verbal similarity judgments in relation to motion events in L2 learners are driven by the learner’s native language (i.e., Chinese), or whether they show signs of restructuring in terms of the target language (i.e., English).

## Encoding Caused Motion Events in English and Chinese

The observation that world languages systematically differ in encoding motion events leads Talmy (1985, 2000) to formulate his motion event typology. In his classification, languages mainly fall into two categories, satellite-framed and verb-framed, depending on where path, the core element of motion, is placed in an utterance. In satellite-framed languages, such as English and German, manner of motion is characteristically encoded in the verb root whilst path is expressed in verb particles (example 1a). In comparison, path is encoded in the grammatically marked category of main verb in verb-framed languages like Spanish and French, whilst manner information is either omitted (by default) or expressed in peripheral devices, such as subordinated clauses or gerunds (example 1b). For analysis of Indo-European languages, this dichotomy is successful, but it does not seem to fit well with those languages in which verbs normally assume a compound form (e.g., Thai, Chinese). In this third type of language, varied aspects of a motion event (e.g., manner, path, and deixis) are simultaneously encoded in compound verbs with equal grammatical status and formal significance (e.g., Slobin, 2004: ‘equipollently framed’ languages; see also example 1c).

(1) Bonny *pushed* the ball *across* the street.
(1) Popi *traverse* la rue *en faisant rouler* le ballon.
  ‘Bonny went across the street while pushing the ball.’
(1) Bonny ba3 qiu2 *tui*1-*guo*4 ma3lu4.
  Bonny ba ball push-across/cross street
  ‘Bonny pushed the ball across the street.’
(1) Bonny *tui*1 zhe qiu2 *guo*4 ma3lu4.
  Bonny push gerund ball cross street
  ‘Bonny, pushing the ball, went across the street.’

It is agreed that English standardly encodes caused motion events in a ‘manner-and-cause verb + path particle’ combination (e.g., *push across* in example 1a above). The typological status of Chinese, however, raises some debates. It is originally classified by Talmy as a satellite-framed language like English, based on the observation that the second element in the compound verb (e.g., *guo*4 ‘across’ in example 1c) is a particle: it belongs to a closed-class set with a limited number of instantiations. Other researchers (e.g., Slobin, 2004) note, however, that the syntactic function of this element is entirely different from that of English particles: it can function as an independent predicate (please compare: ‘He *guo*4 ‘cross’ the street’ vs. ^∗^He across the street). This is partly related to the fact that the Archaic Chinese is a typical verb-framed language and, in the process of a diachronic change, many verbs lose their independent verbal meanings and are weakened into particles/prepositions. In this light, the independent syntactic function of the second element in a Chinese compound verb can be seen as the remnant of this diachronic change (Peyraube, 2006). Despite some controversies, a growing number of studies reveal that, in terms of motion event typology, it is more appropriate to consider Chinese as different from English, i.e., as an ‘equipollent’ language standing midway along a verb-framed/satellite-framed continuum (Li, 1990; Chen and Guo, 2009; Chu, 2009; Beavers et al., 2010; Ji et al., 2011a,b). As a case in point, Ji et al. (2011a,b) conduct a systematic investigation of how Chinese children and adults linguistically describe the type of caused motion events as illustrated in the current experimental design. It is reported that up to 70% of Chinese utterances recruit compound verbs in depicting complex motion events, and these compound forms invariably encode manner, path and (optionally) deixis of motion (e.g., *tui*1-*guo*4 ‘push-cross’ in example 1c). The remaining 30% of responses express path alone in a single verb (*guo*4 ‘cross’ in example 1d) whilst expressing manner (and causality) in a gerund, thus giving rise to a syntactic construction typical of verb-framing languages like French (i.e., the syntactic similarity between examples 1b and 1d).

In English, the marked grammatical category of verb encodes manner of motion (i.e., manner salience), whereas in Chinese, the same verbal domain packages manner and path simultaneously (equal salience of manner and path). Such language differences may have some cognitive implications. Motion event conceptualisation frequently co-occurs (or associates) with ‘manner salience’ in English but with ‘manner–path salience’ in Chinese. According to the psychological theory of associative learning, our event representations emerge from exposure to a number of specific instances of associations, and we construe and categorize specific events by drawing on the association patterns we have encountered (as summarized by Athanasopoulos et al., 2015b, p. 141–142). In fact, the more routine an association becomes, the easier it is to retrieve and utilize it for the purposes of categorisation (Langacker, 2008). Seen this way, in a triads matching task, it is likely that English speakers more frequently utilize ‘manner-salience’ as a basis for their non-verbal similarity judgments (i.e., more manner-matches) whereas their Chinese counterparts recruit ‘manner-salience’ and ‘path-salience’ equally frequently as bases for their decision-making (e.g., comparable proportions of manner- and path-matches). In an L2 context, this mainly concerns the extent to which categorisation is influenced by a conceptual switch from the pattern of ‘manner–path salience’ to ‘manner salience.’

## Conceptualisation of Motion Events in L1 and L2 Speakers

The striking cross-linguistic differences in terms of motion event typology prompts the question of whether motion cognition differs in speakers of languages with opposing properties. Research in this area produces contradictory results with some studies showing almost no obvious effect of language on thought (e.g., Lucy, 1992; Jackendoff, 1996; Gennari et al., 2002; Papafragou et al., 2002; Landau and Lakusta, 2006), while others suggest a clear effect of linguistic relativity (e.g., Naigles and Terrazas, 1998; Boroditsky, 2001; Levinson, 2003; Hohenstein, 2005; Lupyan, 2012; Zlatev and Blomberg, 2015), as examined in varied non-linguistic behavioral tasks, such as memory recognition, categorisation of motion events and preferential looking. For instance, Papafragou et al. (2002) ask English- and Greek-speaking adults and older children to categorize motion events in a triad task similar to ours. Each of the motion events is presented as a series of digital photographs, and all of the events within a triad are laid out in such a way that they can be inspected in parallel by participants. Both 8-year-old children and adults in the two languages behave identically despite substantial differences in the ways that English (satellite-framed) and Greek (verb-framed) linguistically encode motion events. Another experiment (Papafragou and Selimis, 2010) using similar methodologies further reports that only when participants are encouraged to use the linguistic stimuli in the process of event apprehension do they categorize motion events in a language-congruent pattern. When the linguistic relativity hypothesis is tested in the context of bilingual representation, the key issue is whether bilinguals’ conceptualisation differs from that of monolinguals and, if so, how. Findings along this line, as attested in motion event cognition, seem to suggest that learning an additional language can result in conceptual restructuring or shifts in one’s cognitive state (Athanasopoulos, 2009; Brown and Gullberg, 2010; Cook and Bassetti, 2011; Daller et al., 2011; Filipovic, 2011; Pavlenko, 2011; Bylund and Athanasopoulos, 2014; Athanasopoulos et al., 2015b; Flecken et al., 2015; Montero-Melis et al., 2016; Thierry, 2016).

To illustrate, Athanasopoulos et al. (2015b) investigate L2 learners’ motion event cognition in relation to grammatical aspects. In an aspect language such as English, the habitual attention of speakers is directed to the ongoingness of events (e.g., *a baby crawling along the street*) whereas in non-aspect languages like German, people normally pay attention to event endpoints/goals (e.g., *a baby crawling to the zoo*). This difference is explored in English learners of L2 German in a triads matching task in which participants are asked to match a target scene with an intermediate degree of endpoint orientation with two alternate scenes with low and high degrees of endpoint orientation, respectively. The purpose of the study is to find out whether L2 learners whose native language marks grammatical aspects can learn to start paying more attention to event endpoints in the course of acquiring a target, non-aspect language. It is reported that, compared to native speakers (NS) of English, learners of L2 German are more prone to base their similarity judgments on endpoint saliency, rather than ongoingness, primarily as a function of increasing proficiency and length of exposure. These results suggest that English learners of German may have internalized additional perspectives on event construal (i.e., endpoints apart from ongoingness), restructured the frames they have acquired in L1 and shifted their patterns of motion event representation. It is during this process that they have developed a cognitive state that increasingly biases toward the target language.

Athanasopoulos et al.’s (2015b) investigation represents studies focusing on how bilinguals shift toward L2 categorisation patterns as a function of L2 exposure in a relatively long period. Such studies are characterized by comparing behavioral performance between bilingual vs. monolingual groups. Another line of research in bilingual motion event cognition is to assess whether, and how, linguistic cues present in the immediate environment can be recruited by L2 learners to assist them in event categorisation or discrimination. Studies of this type normally concern only bilinguals, who perform behavioral tasks under manipulated linguistic conditions. As a case in point, Montero-Melis et al. (2016) investigate whether L2 priming affects similarity judgments in representing a complex type of caused motion (similar to stimuli in the present study). Specifically, Swedish (satellite-framed language highlighting manner information) adult learners of L2 Spanish (verb-framed language highlighting path information) are asked to read out loud L2 sentences with varying degrees of manner or path salience before arranging motion scenes. Results show that path vs. manner priming affects how participants judge the similarity between events. For example, immediately after reading out L2 sentences highlighting the path dimension, Swedish adult learners tend to arrange motion events on a ‘same-path’ basis. Note that this judging criterion is inconsistent with the typological feature of their native language, thus indicating that when linguistically mediated, cognitive restructuring can be dynamic and context-dependent, and a switch of conceptual representations in bilinguals can be completed in a relatively short time scale.

These findings echo those obtained by Lai and Narasimhan (2015) who examine how bilingual speakers of English and Spanish conceptualize motion events in a forced similarity judgment task. It is reported that bilinguals who describe a motion event in English in the first instance tend to select the event that has the same manner of motion as the target scene significantly more frequently than bilinguals who encode the same event in Spanish prior to judgment. In a similar fashion, Filipovic (2011) tests whether balanced English–Spanish bilingual speakers behave like monolinguals in each of their languages when describing and remembering motion events with different types of manner information. She finds that, regardless of the language used in the experiment, the performance of bilingual participants in the recognition task closely resembles that of the Spanish monolingual speakers. She attributes this to the status of Spanish as a ‘dominant’ language in the life of her bilingual participants.

To summarize, studies of bilingual motion event cognition are in great need of expansion. Previous literature tends to focus on languages with contrasting typological features only (i.e., satellite- vs. verb-framed) and examine, in most cases, spontaneous motion events only [but see Montero-Melis et al. (2016) for an exception]. In this context, the present study expands the pair of languages under investigation to include the non-Indo-European language of Chinese, which is typologically partially similar to English (rather than entirely in opposition to it). This will allow us to test whether the typological similarity between L1 and L2 (i.e., ‘manner-salience’ in both languages) can facilitate L2 learners’ cognitive restructuring in implicit processing.

## Research Questions and Hypotheses

The present study is generally interested in two questions: (a) whether the effect of motion language typology can go beyond language performance, and influence motion conceptualisation of monolingual speakers (as tested in a triads matching task); (b) whether, and how, the motion conceptualisation of L2 learners differs from that of monolinguals. Does the behavioral evidence suggest any significant shift in conceptualisation patterns across proficiencies? This study compares, in the first instance, how different types of speakers (L1 vs. L2 learners; English monolinguals vs. Chinese monolinguals, L2 learners at different proficiencies) evaluate the similarity between motion screens (as indicated by their overt preferences), and the extent to which their decision strategies can be considered language-biased. Further, it investigates whether there is any difference in response latency [as measured by reaction time (RT)] in judgment across different types of speakers, and the extent to which L2 learners’ efficiency in reaction is comparable to that of source (or target) language speakers.

We generate three hypotheses regarding the research questions. First of all, at one extreme, a ‘universal’ prediction might be made in which language-specific influence is only superficial and cannot go deeper into the cognitive level. Following Talmy’s (2000) ‘basic motion scheme’ and the universal cognitive salience of path (vs. manner) in the human mind, different types of speakers may prefer the path-match over the manner-match more frequently in their judgments.

Secondly, at the other extreme, we hypothesize a strong effect of linguistic relativity, in which the behavior of different types of speakers can be largely predicted by relevant language differences. Due to the high ‘codability’ of manner information in the linguistic encoding of motion events in English and its psychological implications (i.e., more habitual attention to manner dimension), we predict that English monolinguals would be more manner-oriented than their Chinese counterparts in more frequently preferring the manner-matches (i.e., less frequently preferring the path-matches) and reacting significantly more quickly in manner-matched decisions than in path-matched choices. Following the main arguments of linguistic relativity, speakers have been trained, since childhood, to think in a way that is largely constrained by their native language; their particular way of thinking should be rather hard to remold in adulthood. We thus predict that L2 learners at low and/or intermediate proficiencies should show behavioral evidence of being more native-language biased; only at an advanced stage of acquisition would it be likely that Chinese learners of L2 English behave in a more target-language biased way.

Previous findings suggest that L2 learners may demonstrate a U-shaped curve across three proficiencies in restructuring their construal of particular types of motion events, for example in studies focusing on a contrast between boundedness vs. unboundedness in aspect. Low-level learners can change their motion event cognitive representations at the initial stage because their system is affected at a general level by statistical learning (similar to the effects of laboratory training). Learners at the intermediate level may seem to revert back to a native-language biased pattern; however, at a fine-grained level of cognition, they undergo a process of suppression (of routinized L1 categorisation pattern) and internalization (of a novel event construal). Only learners of high proficiencies are reported to have shifted completely toward an L2 cognitive pattern (Athanasopoulos et al., 2015b, p. 148–149 for a detailed discussion). The inclusion of three proficiencies in the present study thus aims to reveal clearly any developmental trajectory (linear or non-linear) in cognitive representations of motion events focusing on a comparison between satellite-framedness and equipollently framedness.

Thirdly, similarity judgments of participants will be assessed by both categorical measurement (i.e., overt preferences) and continuous measurement (i.e., RT). The former variable can only answer the dichotomous question of whether language influences non-verbal cognition (i.e., A or B in a forced similarity judgment task), whilst the latter variable allows us to test the degree of differences (if any) in behavior as engendered by language differences. Given the fact that the two languages under investigation are only partially different (rather than entirely opposing), there is a third likelihood, in which the proportion of overt preferences for manner (or path) might not vary significantly across types of participants; any language-biased patterns in behavior will only be evident (if at all) when examined under the lens of RT.

Clearly, decision and RT are two different types of data, and the latter can reveal aspects of processing that are often not available in results from choice response measures. According to Tokowicz and MacWhinney (2005), certain measurements such as event-related potential (ERP) and RT can directly reflect automatic, non-reflective, implicit responses to stimuli. We thus reason that any differences in RT between manner- vs. path-match in our study would reflect online implicit processing, and that overt manner- or path-matched choices would reflect primarily explicit processing. Previous literature suggests that there is often a divergence between explicit and implicit measures of L2 learning, which may be due to the behavioral task demand.

To illustrate, Li et al. (1993) investigate how participants interpret sentences in a language without inflections (i.e., Chinese) by using other types of linguistic cues (such as word order and noun animacy). Chinese L2 learners hear a sentence played back on a speaker and simultaneously see on the computer screen a pair of pictures that correspond to the two objects described in the sentence. They are then asked to decide which of the two objects in the pictures is the doer of the action in the sentence by indicating their choice on a button box. It is reported that although the final choice decisions for two different sentences may be the same, the amount of time it takes to reach the same decision is very different, showing clear effects of linguistic cues with varying degrees of strength in the interpretation process. Although the current study focuses on motion animations, not sentence interpretation, we believe that the two tasks recruit the same mental format that codes the interpretation, rather than the perceptual properties of sentences or pictures/videos. In other words, both tasks involve thought processes that are cognitive in nature and deal with interpreted knowledge [see Clark and Chase (1972) for a detailed discussion]. In this light, we include in our study the continuous measurement of RT and compare it with the behavioral data with the aim of providing a more sensitive method for measuring implicit processing.

## Methodologies

### Participants

One hundred and sixty adult students participated in the study; all were university or senior high school students. They were divided into five groups with 32 gender-balanced students per group. Chinese monolingual speakers came from a technical school of the Shandong Province in China and English NS were recruited from King’s College, London. The three groups of adult Chinese learners of L2 English consisted of students from Shenzhen University, China. Permission to recruit and advertise the study was granted by the schoolmaster of the technical school in Shandong China, the Committee of Research Ethics of King’s College, London and the Committee of Academic Affairs of Shenzhen University, respectively. Informed consent forms and demographic information sheets were collected from participants prior to the start of the experiment. All students received a monetary reward for their participation.

The proficiency levels of L2 learners (low, intermediate and advanced) were determined by their test scores in the English Language Proficiency Tests, administered twice a year by the Ministry of Education, China. This formal measure of general proficiency in the English language distinguishes three levels: Band 4, Band 6, and Band 8. There are separate test papers for the three bands, with identical test paper design and scoring system, but different proficiency requirements in various aspects, such as listening, reading, writing, understanding and Chinese-English translating. All L2 learners in our study had taken the aforementioned tests in the 6 months before the experiment. The low proficiency level learners are students who had only passed the Band 4 test; the intermediate level students had passed the Band 6 test; those who had passed the Band 8 proficiency test were categorized as advanced learners and read L2 English as part of their degree in English language or literature. All L2 students (Mean age = 22.3 years) had similar learning backgrounds, with systematic English input from around the age of 12. They all acquired English in a predominately Chinese-speaking community and their English input mainly came from classroom teaching (see **Table 1**, below).

**Table 1:** Groups of participants in the study.

Group ID	Age (*M and *SD*)*	Proficiency level	Proficiency score (*M* and *SD*)	Length of L2 exposure	Number of participants
CHNS	19.30 (0.97)^1^	Chinese NSs	N/A	N/A	32
L2-Low	20.28 (1.76)	Elementary learners of English	70.97 (6.32)	7.19 years	32
L2-Medium	21.16 (1.14)	Intermediate learners of English	69.23 (6.23)	8.06 years	32
L2-High	24.77 (2.06)	Advanced learners of English	70.89 (6.70)	11.61 years	32
ENNS	26.00 (5.17)	English NSs	N/A	N/A	32


*Note: L2-Low: second language learners of English (low proficiency); L2-Medium: second language learners of English (intermediate proficiency); L2-High: second language learners of English (advanced proficiency); ENNS: monolingual English native speakers, and CHNS: monolingual Chinese native speakers.*

*^1^The group of monolingual speakers of Chinese does not match exactly with other groups in age and educational background (i.e., they are technical school students with a mean age of 19.30). This is because it is virtually impossible to recruit entirely and completely monolingual Chinese native speakers who are also educated to university level. We have made sure that the senior technical school students we recruited have only basic to lower knowledge of English due to the course design in their school.*

### Materials

Forty-eight short video clips (5 s each) were used as our stimuli, each depicting a caused motion event, in which both manner (coupled with cause) and path were presented as equally salient. These stimuli conformed to previous models of caused motion developed by Hickmann et al. (2009): both presented a specific type of caused motion, in which an agent carried out a specific action to an object, which changed its location due to the external force; meanwhile, the agent accompanied the moving object (by *walking*) throughout its course of movement. All stimuli illustrated six specific types of manner (*pushing*/*pulling*, *rolling*/*sliding*, *throwing*/*kicking*) and eight types of path: verticality (*up* and *down*), boundary crossing (*across* and *into*), deixis (*toward* and *away from*) and parallel to, or encircling, the ground (*along* and *around*). To illustrate, the target scene in triad 2 in Appendix A depicted a boy (Bonny) pulling a treasure bag up a pyramid, the bag sliding up the pyramid and the boy accompanying the bag all the way to the top of the pyramid (see also Appendix B for illustration).

A total of 48 motion video clips were organized into a set of 16 triads: 16 targets and 32 alternates (two for each of the target events). Target and alternate videos appeared for 5 s each and were then followed by 1 s of black screen. The task lasted approximately 6 min in total. All stimuli were arranged into two randomized orders: A and B (A reversed). These orders were counterbalanced across participants within group. The presentation position of manner-matched vs. path-matched video clips (left side or right side of the screen) was counterbalanced across stimuli in a given order.

Within a triad, the target video clip depicted the boy performing a specific action which caused the movement of an object (e.g., pulling a treasure bag up the pyramid), while the two alternate video clips showed the same boy involved in similar actions with changes in either manner or path. In the path-match alternate, the path of motion remained the same while the manner of motion was changed (e.g., *pushing* a treasure bag up the pyramid), while in the manner-match alternate, the path varied with manner kept the same (e.g., pulling a treasure bag *into* the pyramid). In order to direct the participants’ attention to the similarity between actions rather than anything else, all stimuli involved the same boy with the same clothing. In each triad, the background scenery for motion was also kept uniform across the target and two alternates. A complete list of stimuli is given in Appendix A.

### Procedures

#### Pre-test

A pre-test for perseveration was administered prior to the testing session^1^. Participants were shown five triads of static pictures showing ordinary objects, such as pigeons, flowers, and bananas. Within each triad, the target object was placed in the center of a piece of paper, while two alternates were shown side-by-side on a separate page, each differing from the target by either size only (e.g., a bunch of big bananas vs. a bunch of small bananas) or color only (a bunch of yellow bananas vs. a bunch of green bananas). The participants were asked to identify which of the two alternates was most like the target object. If participants had chosen alternates from only one side of the page for all five selections (none did), they would have been considered perseverative and thus excluded from the study.

#### Testing Session

A single female experimenter tested participants individually. The participants were invited to watch video clips displayed on a MacBook Pro and asked to signal their judgments of similarity between motion scenes by pressing one of two keys on the keyboard, which were covered with white stickers (i.e., no linguistic labeling): ‘A’ and ‘L,’ respectively. A training item (target: a boy walking down stairs; manner-alternate: a boy *jumping* down stairs; path-alternate: a boy walking *up* stairs) preceded the test phase. The participant was asked to observe the video clips carefully and decide quickly which of the two alternates was most like the target. They were instructed to indicate their choices by pressing a specific key on the keyboard. This training item mirrored the task that the participants would perform in the subsequent test phase and aimed to direct the attention of participants to the overall similarity between motion events *per se*. Sixteen triads of video clips were played to the participants on the laptop screen through the stimulus presentation software ‘SuperLab 4.5,’ which generated, at the end of each testing session, a file containing information such as the participant’s choices (manner-match or path-match) and his or her RT.

The stimuli were played in a synchronized series with the target videos playing first in the center of a screen, followed by two simultaneous alternate videos placed side-by-side on the same screen. There was a black screen of 0.5 s between a target and two alternates within each triad, and a 1 s black screen between triads. The participant was instructed to view the stimuli and make his or her decisions as quickly as possible. Audio stimuli accompanying the video were: “Target: This is X.” “Alternates: Which one is most like X?”^2^ In order to render the current study a truly ‘non-linguistic’ one, a verbal interference task was utilized in which a random sequence of numbers were broadcast to the participants throughout the testing phase, with the aim of preventing them from subconsciously verbalizing motion scenes during the process of their decision-making (see similar ‘number-shadowing’ tasks in Gennari et al., 2002).

### Coding

The dataset was measured by two variables. The categorical variable refers to overt selections (either manner-match or path-match) made by the participants. These choices were determined by given keys (A or L) the participant had pressed while viewing the motion stimuli. The other type of RT variable is continuous in nature and aims to test the degree of differences (if any) across types of participants. The RT for a given stimulus was calculated from the time between the onsets of presentation of alternate videos in a triad until their completion (including a 1 s black screen); theoretically, therefore, the longest RT is 6,000 milliseconds (ms). The data was first cleaned by excluding physically impossibly short values (key pressed down within 200 ms of stimulus onset). For extremely long values, screening for outliers was performed by removing all observations that were more than two standard deviations (SD) from the mean.

## Results

This section reports findings in relation to two main questions: (a) whether participants’ behavioral responses vary significantly with group (i.e., CHNS, L2-Low, L2-Medium, L2-High, ENNS) and/or with preference type (i.e., manner-match and path-match; see “Participants” sub-section); (b) whether the overall RT to motion stimuli varies significantly as a function of participant group and/or preference type (see “Materials” sub-section). Depending on specific questions asked, statistical tests, such as two way mixed analysis of variance (ANOVA), were utilized to explore relevant datasets.

### Number of Manner- and Path-Matched Judgments across Five Participant Groups

**FIGURE 1:**
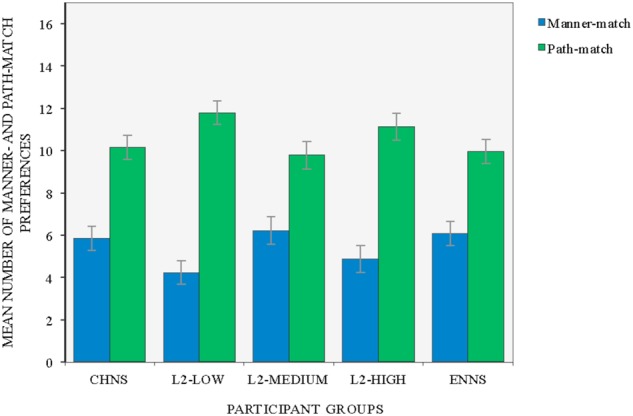
Mean number of manner-matched and path-matched preferences across five participant groups (error bars indicate mean ± SE).

The preferences of the participants were decided according to the specific key (A or L on the keyboard) they signaled while judging the similarity between motion screens. The data was thus represented as falling into one of the two major strategies: the manner-match or the path-match. The mean was calculated by recording the number of matches out of 16 individuals in a group. **Figure 1**, below, presents the mean number of both manner-match and path-match preferences across five participant groups (CHNS, L2-Low, L2-Medium, L2-High, and ENNS). There seemed to be a shared tendency for the path-match over the manner-match across groups.

A two-way mixed ANOVA with participant group (CHNS, L2-Low, L2-Medium, L2-High, and ENNS) as the between subjects factor, and preference type (manner-match, path-match) as a within-subjects factor revealed a main effect of preference only, *F*(4,155) = 90.204, *p* < 0.001, η^2^ = 0.368. The total mean number of path-matches (*M* = 10.556) significantly exceeded that of manner-matches (*M* = 5.444, *p* < 0.001). No interaction effect between group and preference type was observed, *F*(4,155) = 2.048, *ns*^3^. A closer look at the data further indicated that the mean number of path-matched preferences reached significantly above average (i.e., 8 out of 16 matches) in all five groups: CHNS: *M* = 10.16 (*SD* = 3.254), 95% CI [8.98, 11.33]; L2-Low: *M* = 11.78 (*SD* = 3.160), 95% CI [10.64, 12.92]; L2-Medium: *M* = 9.78 (*SD* = 3.705), 95% CI [8.45, 11.12]; L2-High: *M* = 11.13 (*SD* = 3.617), 95% CI [9.82, 12.43] and ENNS: *M* = 9.94 (*SD* = 3.252), 95% CI [8.76, 11.11]. These results disconfirmed one of our hypotheses regarding a possible strong effect of linguistic relativity; English monolinguals did not prefer the path-match significantly less frequently than their Chinese counterparts.

A different perspective was taken on these results by conducting a by-item analysis on the choices. A two-way repeated measures ANOVA with group (CHNS, L2-Low, L2-Medium, L2-High, and ENNS) and preference type (manner-match, path-match) as two within-items factors revealed, first of all, a main effect of preference, *F*(4,60) = 8.591, *p* = 0.01, $\eta_{p}^{2}$ = 0.364. The total mean number of path-matches (*M* = 21.125) significantly exceeded that of manner-matches (*M* = 10.875, *p* < 0.01). Meanwhile, the analysis showed a significant interaction between group and preference, *F*(4,60) = 8.266, *p* < 0.001, $\eta_{p}^{2}$ = 0.355. Pairwise comparisons with Bonferroni correction confirmed that the differences in the total mean number of path-matches vs. manner-matches were significant in monolingual speakers of Chinese (CHNS: mean difference = 8.625, *p* = 0.033, $\eta_{p}^{2}$ = 0.270), L2 learners of low proficiency (L2-low: mean difference = 15.125, *p* = 0.001, $\eta_{p}^{2}$ = 0.541) and those at the advanced level of acquisition (L2-High: mean difference = 12.500, *p* = 0.001, $\eta_{p}^{2}$ = 0.525). There was a trend toward a significant difference in the total mean number of path-matches vs. manner-matches in L2 learners of medium proficiency (L2-Medium: mean difference = 7.25, *p* = 0.064) and monolingual speakers of English (ENNS: mean difference = 7.75, *p* = 0.061). Thus, the two sets of analyses (i.e., by participant and by item) roughly converge on differences arising between the path-match and the manner-match.

### RT in Manner- and Path-Matched Judgments across Five Participant Groups

In this sub-section, we investigated whether the overall RT to video clips varied significantly with participant group and/or preference type. A by-participant mixed ANOVA was performed in the first instance, which was followed by a by-item repeated measures ANOVA.

**FIGURE 2:**
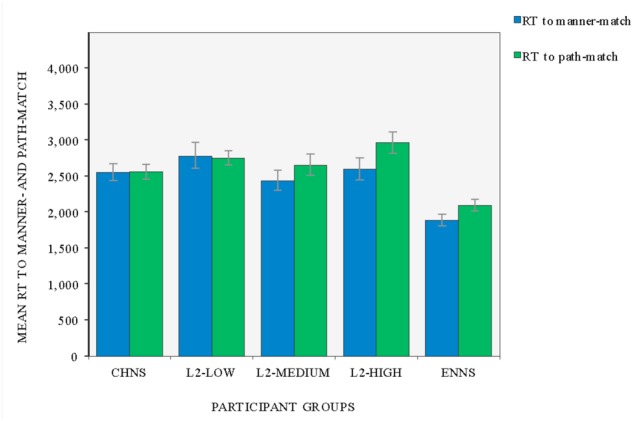
The mean RT (in ms) to manner- vs. path-match across participant groups (error bars indicate mean ± SE).

A two-way mixed ANOVA was first conducted with group (CHNS, L2- low, L2-Medium, L2-High, and ENNS) as the between subjects factor and preference type (manner-match, path-match) as a within subjects factor. It revealed a statistically significant main effect for participant group, *F*(4,155) = 6.853, *p* < 0.001, $\eta_{p}^{2}$ = 0.150, as well as a statistically significant main effect for preference type, *F*(1,155) = 11.413, *p* = 0.001, $\eta_{p}^{2}$ = 0.069. Furthermore, a statistically significant interaction between group and preference type was attested, *F*(4,155) = 2.756, *p* = 0.030, $\eta_{p}^{2}$ = 0.066 (see **Figure 2**).

Pairwise comparisons with Bonferroni adjustment were used to further examine the interaction between participant group and mean RT to manner- vs. path-match. These analyses indicated that differences in the mean RT to manner- vs. path-matched conditions were statistically significant for monolingual speakers of English (ENNS: mean difference = -210, *p* = 0.033, $\eta_{p}^{2}$ = 0.029), L2 learners of high proficiency (L2-High: mean difference = -368, *p* < 0.001, $\eta_{p}^{2}$ = 0.084) and those at the intermediate level (L2-Medium: mean difference = -214, *p* = 0.029, $\eta_{p}^{2}$ = 0.030). However, the RT to manner- vs. path-matched conditions was approximately equivalent for monolingual speakers of Chinese (CHNS: mean difference = -4, *ns*) and L2 learners at the initial stage of their acquisition (L2-Low: mean difference = 28, *ns*; see also **Table 2**).

**Table 2:** RT (in ms) in manner-matched and path-matched conditions across participant groups.

Participant groups	Mean RT in manner-match	Mean RT in path-match
CHNS	2553 (*SD* = 674)	2557 (*SD* = 602)
L2-Low	2780 (*SD* = 1028)	2752 (*SD* = 559)
L2-Medium	2437 (*SD* = 784)	2652 (*SD* = 838)
L2-High	2593 (*SD* = 858)	2961 (*SD* = 832)
ENNS	1883 (*SD* = 477)	2093 (*SD* = 472)


As mentioned earlier, the two-way mixed ANOVA confirmed that, generally, English speakers were significantly quicker in responding to motion stimuli in their similarity judgments, as compared to all other four groups (i.e., the main effect of group). A closer examination of the dataset via multiple comparisons with Bonferroni correction further revealed that in making manner-matched choices, monolingual speakers of English were significantly quicker than their Chinese peers (mean difference = -670, *p* = 0.008), as well as the three L2 groups (ENNS vs. L2-low: mean difference = -897, *p* < 0.001; ENNS vs. L2-Medium: mean difference = -555, *p* = 0.052, and ENNS vs. L2-High: mean difference = -710, *p* = 0.004). By contrast, in opting for path-matched motion scenes, monolingual speakers of English did not differ significantly from their Chinese counterparts (mean difference = -464, *ns*), though they still reacted quicker as compared to the learner groups (ENNS vs. L2-low: mean difference = -659, *p* = 0.001; ENNS vs. L2-Medium: mean difference = -559, *p* = 0.012, and ENNS vs. L2-High: mean difference = -868, *p* < 0.001). Such a contrast in RT between the two groups of monolingual speakers is of particular importance in indicating that the overall quicker RT of English monolinguals can be attributed to their even quicker reaction to manner-matches.

Secondly, an additional by-item analysis was further performed on the RT data to investigate whether the interaction effect between participant group and preference type remains systematic across individual items. A two-way repeated measures ANOVA with group (CHNS, L2-Low, L2-Medium, L2-High, and ENNS) and preference type (manner-match, path-match) as two within-items factors showed a main effect of group, *F*(4,60) = 40.119, *p* < 0.001, $\eta_{p}^{2}$ = 0.728, as well as a marginally statistically significant effect of preference type, *F*(1,15) = 4.262, *p* = 0.057, $\eta_{p}^{2}$ = 0.221. Meanwhile, the test revealed a significant interaction between group and preference, *F*(4,60) = 2.901, *p* = 0.029, $\eta_{p}^{2}$ = 0.162. Pairwise comparisons with Bonferroni correction further revealed that the differences in mean RT between manner- vs. path-match were significant in monolingual speakers of English (ENNS: mean difference = -336, *p* = 0.011, $\eta_{p}^{2}$ = 0.358) and L2 learners of high proficiency (L2-High: mean difference = -433, *p* = 0.036, $\eta_{p}^{2}$ = 0.262). However, no difference was detected for the group of monolingual speakers of Chinese (CHNS: mean difference = 0.128, *ns*), as well as L2 learners at low and intermediate levels (L2-Low: mean difference = 27, *ns*; L2-Medium: mean difference = -283, *ns*).

In summary, our by-participant and by-item analyses converge on an observation that the differences in RT to manner-match vs. path-match vary significantly, depending on participant group. Great discrepancies were attested between typologically different L1s (English vs. Chinese), different learner types (monolinguals vs. L2 learners), and across different proficiencies amongst L2 learners (Low and/or Medium vs. High). Monolingual speakers of English reacted significantly quicker to the manner-match than to the path-match, whereas their Chinese counterparts used an approximately equal amount of time in making manner- and path-matched decisions. As for L2 learners, they seem to achieve a target language-biased cognitive pattern only at a relatively advanced stage of acquisition.

## Discussions and Conclusions

The current study investigates how Chinese learners of English at different proficiencies conceptualize motion in a triads matching task, as compared to monolingual speakers of English and Chinese. Two main questions are asked: (a) whether the effect of motion language typology can go beyond language performance and influence non-verbal event categorisation of monolingual speakers; (b) whether, and how, the non-verbal event categorisation of L2 learners differs from that of monolinguals and, further, whether there are any significant shifts in motion cognition pattern across proficiencies, as suggested by the behavioral evidence. Overall, our two sets of analyses (i.e., categorisation preferences and RT data) produce seemingly conflicting results. In terms of choice response, both monolinguals of different languages and L2 learners at varied proficiencies prefer the path-matched screens in judgment. However, in terms of RT, English monolinguals, as well as medium and/or high proficiency L2 learners, respond more efficiently to manner-matched (vs. path-matched) motion stimuli than Chinese monolinguals, and low-level L2 learners. It merits mentioning that such significant differences in RT across five groups are obtained in a truly ‘non-linguistic’ context (i.e., the ‘number-shadowing’ procedure). Given that the effect of linguistic relativity is normally canceled in verbal interference tasks as revealed in some previous studies (e.g., Gennari et al., 2002; Papafragou et al., 2002; Athanasopoulos et al., 2015a), the set of findings regarding the RT data is of particular significance in suggesting that the effects of language may be strong enough to lead to variations in non-verbal categorisation of motion events even in the presence of verbal interference.

The first question arising from the above reported findings may be why Chinese and English monolinguals did not show any significant variations in their choice response, despite the striking differences in linguistic encoding of motion events between these two languages? Such an observation may be interpreted from different perspectives. One possibility, as suggested by Lupyan (2012), is that describing events constitutes an inherently more complex form of linguistic labeling than, say, object naming. Therefore, any widely attested differences in motion description across world languages tend to be probabilistic rather than categorical, showing great within-language variability. As a case in point, French is traditionally and standardly considered as a verb-framed language, but recent studies reveal that apart from expressing path of motion in the verb, French can also express path in a prefix demonstrating a satellite-framed pattern with high degree of productivity (Kopecka, 2006, p. 85–91). The implication of such observations is that due to this variability within language, habitual language experience “might not bias speakers of different languages to distinct event components in a categorical way, which, in turn, would limit the cross-linguistic differences one should expect in non-verbal tasks” (see Montero-Melis et al., 2016, p. 642 for a detailed discussion).

Note that our results regarding the choice data are inconsistent with findings of some previous studies using similar triads matching tasks in relation to motion events. To illustrate, Athanasopoulos et al. (2015b) report that English (an aspect language) focuses on the ‘ongoingness’ of events whereas German (a non-aspect language) emphasizes on the ‘end point/goal’ of motion. Such a crosslinguistic difference is found to influence the non-verbal event categorisation of NSs of the two languages. German monolinguals tend to base their judgments on endpoint saliency whereas their English peers prefer to select on the basis of ‘ongoingness.’ The discrepancy between present results for the choices and those in the study of Athanasopoulos et al. (2015b) may be attributable, in the first instance, to the absence of verbal interference in the latter study in which participants may have subconsciously used the linguistic clues to aid their judgments^4^.

A more important reason for the discrepancy, however, might be related to the degree of differences between the two languages under comparison in given aspects. In Athanasopoulos et al.’s (2015b) study, there seems to be a black-and-white contrast between English and German in terms of grammatical aspect [i.e., (+aspect) vs. (-aspect)]. Such a stark contrast does not hold between Chinese and English (and in general, between equipollently- and satellite-framed languages) in terms of manner and path salience. In both languages, the manner information is highlighted in the marked grammatical category of verb (in verb root in English and in compound verb in Chinese). The path dimension is characteristically encoded in both languages as well though its salience is accentuated in varying degrees: it is encoded in the grammatically marked category of verb in Chinese but outside the verbal domain in unmarked categories of particles and prepositions in English. Put another way, manner and path salience is essentially a matter of degree (rather than an ‘either manner or path’ distinction) in the current study. In English, although manner is linguistically presented as more salient than path, such relative salience is not sufficiently strong to exert a categorical impact on non-verbal behavior, say, leading to a preference for the manner-matched scenes amongst English monolinguals. Given that path (rather than manner) is the only indispensable and universally salient ingredient for any motion event [see Talmy’s (2000) ‘Basic motion scheme’], it is little wonder that Chinese and English monolinguals behave similarly in their overt choices (i.e., a shared preference for the path-match) in the current experimental scheme.

The above discussions highlight the importance of introducing the RT data into our analysis: they may be more sensitive to language effects that are too probabilistic to detect in the choice response in a forced similarity judgment context. In hindsight, the adoption of RT in measuring the implicit processing in similarity judgments is particularly useful to our study. Overt choices (i.e., manner- or path-match) reveal information about what decisions participants make, and how often they make it, whilst the RT provides information about the speed with which participants render a judgment. Given that the two sets of measurements produce findings that are inconsistent with each other, the RT data aids us in revealing aspects of processing that are not readily available in choice results.

Reaction time is traditionally defined as a measure of the time it takes people to retrieve information from memory (Collins and Quillian, 1969). Questions arise as to what information has been retrieved in context, as presented in the current experimental design. Given the universal cognitive salience of path for motion event conceptualisation [Talmy’s (2000) ‘basic motion scheme] and the high ‘codability’ of manner in both Chinese and English, it is highly likely that both manner and path dimensions are retrieved by participants in the judgment process. The real difference between monolingual speakers of Chinese and English lies in the way that retrieved information has been processed prior to rendering a judgment. Given that manner and path receive equal salience in Chinese, it seems plausible to assume that these two dimensions are processed in a ‘parallel’ fashion, that is, Chinese monolinguals weigh simultaneously the salience of manner vs. path before they arrive at a conclusion. By contrast, NSs of English may have attended to the manner dimension for judgment in the first instance. In actual behavior, although they mostly prefer the path-match, when they do choose the manner-match, they are responding particularly quickly. In cases where aspects of the motion stimuli are deemed not sufficiently supportive of a judgment based on manner-salience, English monolinguals then turn to the path dimension for further processing. In other words, English monolinguals may probably deal with the retrieved information in a ‘sequential’ way. Seen in this light, a ‘negation’ phase seems to exist among English monolinguals between their initial manner-matched evaluation and the subsequent path-matched judgment. According to Collins and Quillian (1969, p. 502), a typical model for Negation Time consists of three phases: reading time (viewing time in our case), encoding time and comparison time. It is reasonable to hypothesize that in reaching a path-matched decision, an additional process of negation may have prolonged the encoding time, thus resulting in the great discrepancy in RT to manner- vs. path-matched videos in English monolinguals.

As for L2 learners, our study suggests that those participants of medium and high proficiencies can reconstruct their motion cognition pattern biased toward the target language in online processing, although the more novice learners still show a native-language biased cognitive mode. Slobin (1996, p. 89) predicts, in his ‘thinking for speaking’ hypothesis, that learning a second language basically means acquiring an alternative way of thinking, and the L1 ‘thinking for speaking’ pattern, which is ingrained from childhood, is “exceptionally resistant to reconstructing in adult second language acquisition.” There is already an abundant literature concerning the L2 acquisition of motion description, mostly confirming that L2 learners are able to shake off the constraints of their L1 linguistic pattern and get acclimatized to the target language-biased pattern whilst speaking an L2. This even occurs when L1 and L2 are typologically opposing or the linguistic pattern to acquire involves complicated and advanced language skills, such as syntactic construction and discourse organization (Cadierno, 2004; Cadierno and Ruiz, 2006; Ji and Hohenstein, 2014a,b, to name a few). Taken together, our findings seem to suggest that when engaged in online language-recruiting activities (e.g., speaking, listening, and translating) or in non-verbal behavioral tasks, an individual’s motion event cognition pattern might not be as resistant to remolding as is previously proposed.

Having said that, one should bear in mind that in our particular experimental context, the languages in comparison are at least partially similar in terms of motion event typology (i.e., both have satellite-framing properties) and motion event representation (i.e., both focus on manner-salience). Therefore, it sounds plausible to assume that the L2 learners in our task do not have necessarily completed a process of conceptual convergence or switch; instead, in our case, in which the L2 cognitive pattern for motion (i.e., manner-salience) constitutes a subset of the L1 motion conceptualisation pattern (i.e., both manner and path salience), L2 learners may have simply activated the relevant part of the conceptualisation pattern in their native language in order to utilize it in their implicit processing.

In summary, our findings highlight the complexity of research into motion (and space in general), language and mind. Much ground has yet to be covered in research in an L2 context. When interpreting the role of language in spatial conceptualisation, future studies should take into account variations along a number of dimensions, such as the experimental stimuli used (static pictures vs. dynamic video clips), the nature of motion events investigated (spontaneous vs. caused), the degree of typological similarity/difference between languages under examination, and the measure used to tap into the cognitive state of a mind (e.g., choice response, RT, ERP).

## Ethics Statement

This study was carried out in accordance with the recommendations of ‘The academic Committee for Humanities and Social Sciences of Shenzhen University’ with written informed consent from all subjects. All subjects gave written informed consent in accordance with the requirement of Shenzhen University. The protocol was approved by the ‘The academic Committee for Humanities and Social Sciences of Shenzhen University.’

## Author Contributions

The author confirms being the sole contributor of this work and approved it for publication.

## Conflict of Interest Statement

The author declares that the research was conducted in the absence of any commercial or financial relationships that could be construed as a potential conflict of interest.

## Footnotes

1 The term “perseveration” can be roughly understood as some form of response repetition or the inability to undertake set shifting (e.g., changing of behavior, activities, etc.; see Helm-Estabrooks, 2004 for a detailed explanation). In our particular experimental set-up, a “perseverative” participant would tend to choose the target motion scene from only one side of the screen. Since manner- and path-matched video clips alternate at the left (or right) position of the screen, this would systematically lead to an equal number of selections for manner- and path-matched scenes, thus failing to show any particular preference for either the manner or the path dimension. Therefore, we followed Hohenstein (2005) in the experimental procedures by excluding potential “perseverative” participants from the study.

2 In the present study, the language of instruction (i.e., in the audio stimuli) for the learner groups is English, whereas the language context (on informed consent forms and demographic information sheets) is Chinese. Some recent studies suggest that the language involved in a non-verbal task may exert a context-bound and transient effect on bilingual cognition (but see Filipovic, 2011 for different findings). For instance, Athanasopoulos et al. (2015a) report that fluent German–English bilinguals categorize motion events according to the grammatical constraints of the language in which they operate. Other researchers have different observations suggesting that if bilingual groups have access to both languages during the task, any effects induced by language context may be effectively wiped out (see Montero-Melis et al., 2016, p. 640–642 for a detailed discussion).

3 The effect of the ‘group’ variable was not computed in two-way ANOVAs in this sub-section because there were no missing values in relevant datasets and the total number of preferences (i.e., both manner- and path-matches) remained as a constant across participants, groups and items.

4 In a similar study conducted by Athanasopoulos et al. (2015a), the crosslinguistic differences between English and German in terms of grammatical aspect are reported to disappear in non-verbal event categorisation in the presence of verbal interference. Participants of both language groups show a preference for motion ongoingness, probably because in both the target scene and the ongoingness alternates, the endpoint is not reached (see Athanasopoulos et al., 2015a, p. 523 for a detailed discussion).
