# Supplementary material for: Motion Event Similarity Judgments in One or Two Languages: An Exploration of Monolingual Speakers of English and Chinese vs. L2 Learners of English
Source: Front Psychol. 2017 Jun 7;8:909. doi: 10.3389/fpsyg.2017.00909 (PMC5461365; doi:10.3389/fpsyg.2017.00909)
Supplement: Supplementary file 1 [file Data_Sheet_1.docx]

**Appendix A** A description of sixteen triads of video clips in similarity judgments

| Item | Target | Manner-match | Path-match |
| --- | --- | --- | --- |
| training | Walk down stairs | Walk up stairs | Jump down stairs |
| 1 | Kick balloon up hill | Kick balloon down hill | Throw balloon up hill |
| 2 | Pull bag up pyramid | Pull bag into pyramid | Push bag up pyramid |
| 3 | Roll ring down dune | Roll ring away from dune | Push ring down dune |
| 4 | Pull hay up ladder | Pull hay around ladder | Roll hay up ladder |
| 5 | Kick ball into puddle | Kick ball out of puddle | Roll ball into puddle |
| 6 | Kick ball across slide | Kick ball down slide | Push ball across slide |
| 7 | Push boat out of lake | Push boat towards lake | Pull boat out of lake |
| 8 | Drag toy car across ice | Drag toy car around ice | Slide toy car across ice |
| 9 | Push log towards campfire | Push log away from campfire | Roll log towards campfire |
| 10 | Roll sack towards escalator | Roll sack up escalator | Slide sack towards escalator |
| 11 | Pull suitcase away from tent | Pull suitcase into tent | Slide suitcase away from tent |
| 12 | Pull tyre towards rails | Pull tyre along rails | Throw tyre towards rails |
| 13 | Roll basketball along chairs | Roll basketball around chairs | Throw basketball along chairs |
| 14 | Push balloon around slide | Push balloon up slide | Kick balloon around slide |
| 15 | Pull box along tunnel | Pull box out of tunnel | Kick box along tunnel |
| 16 | Drag barrel around table | Drag barrel away from table | Roll barrel around table |

**Appendix B** An illustration of stimulus triad no. 2 in similarity judgment

**
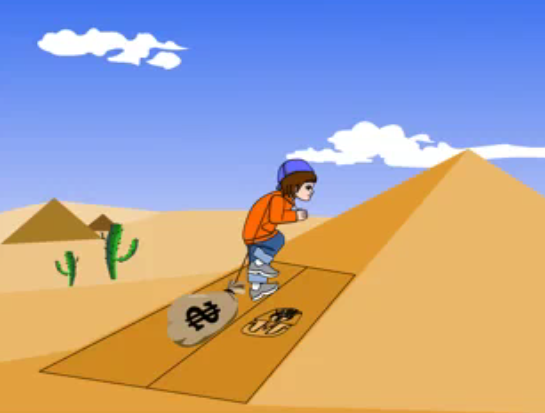
**

2a. Pulling treasure bag up pyramid

**
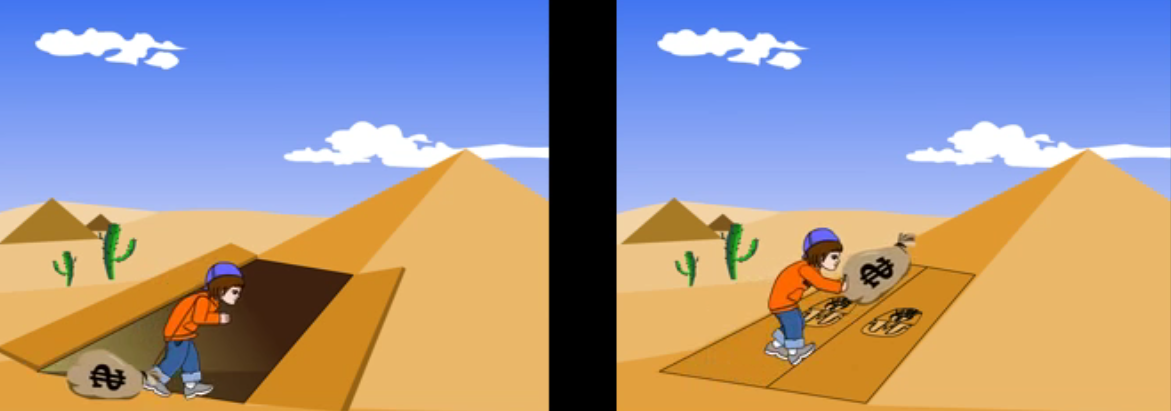
**

2b. Pulling treasure bag *into* pyramid 2c. *Pushing* treasure bag up pyramid
